# Hormonal regulation of root hair growth and responses to the environment in Arabidopsis

**DOI:** 10.1093/jxb/eraa048

**Published:** 2020-01-29

**Authors:** Kris Vissenberg, Naomi Claeijs, Daria Balcerowicz, Sébastjen Schoenaers

**Affiliations:** 1 Integrated Molecular Plant Physiology Research, Department of Biology, University of Antwerp, Antwerp, Belgium; 2 Plant Biochemistry and Biotechnology Lab, Department of Agriculture, Hellenic Mediterranean University, Stavromenos PC, Heraklion, Crete, Greece; 3 University of Warwick, UK

**Keywords:** Arabidopsis, environment, hormone signalling, molecular pathway, root hairs, soil composition

## Abstract

The main functions of plant roots are water and nutrient uptake, soil anchorage, and interaction with soil-living biota. Root hairs, single cell tubular extensions of root epidermal cells, facilitate or enhance these functions by drastically enlarging the absorptive surface. Root hair development is constantly adapted to changes in the root’s surroundings, allowing for optimization of root functionality in heterogeneous soil environments. The underlying molecular pathway is the result of a complex interplay between position-dependent signalling and feedback loops. Phytohormone signalling interconnects this root hair signalling cascade with biotic and abiotic changes in the rhizosphere, enabling dynamic hormone-driven changes in root hair growth, density, length, and morphology. This review critically discusses the influence of the major plant hormones on root hair development, and how changes in rhizosphere properties impact on the latter.

## Teaser

This review presents a molecular framework for how phytohormones regulate normal root hair development and how this is affected by changes in the rhizosphere, to enable greater understanding of the specialized functions of root hairs and their developmental and environmental plasticity.

## Introduction

Roots provide structural anchorage and access to water and nutrients, which is vital to plant survival. In addition, they form the site of symbiotic interactions with soil-living microorganisms (Haling *et al.*, 2013; Grierson *et al.*, 2014). As roots encounter heterogeneous soils in which nutrients and water are not equally distributed, their root system architecture is constantly modified. Dynamic modification of root hair growth, length, density, and morphology allows the plant to meet its nutrient demand in a variety of soil conditions, with the aim of creating an optimal soil-sampling root volume (Forde and Lorenzo, 2001; López-Bucio *et al.*, 2003; Morris *et al.*, 2017). The importance of root hairs is illustrated by the fact that overall plant fitness is negatively affected in several loss-of-function root hair mutants when grown in challenging soil conditions (Wu *et al.*, 2007; Miguel *et al.*, 2015). Consequently, dynamic root hair morphogenesis is a key trait for improving the acquisition of essential nutrients in heterogeneous soils. Therefore, a better understanding of the root hair developmental pathway is crucial. Various studies, mostly based on the model organism Arabidopsis, have led to a better understanding of the genetic and molecular cascade that lies at the base of root hair development (Balcerowicz *et al.*, 2015; Schoenaers *et al.*, 2017).

Nevertheless, it is far from clear how different plant hormones feed into this genetic framework. In addition, many environmental conditions influence different stages of root hair morphogenesis, in particular nutrient availability (phosphorus, nitrogen, iron, etc.), water deficiency, and microbe interaction. Currently, it is not fully understood how these different signals are integrated by the plant root, and how they fine-tune root hair development. Here, we review the current state-of-the-art regarding hormonal regulation and environmental impact on root hair development.

## General hormonal regulation of root hair development

**Fig. 1.**
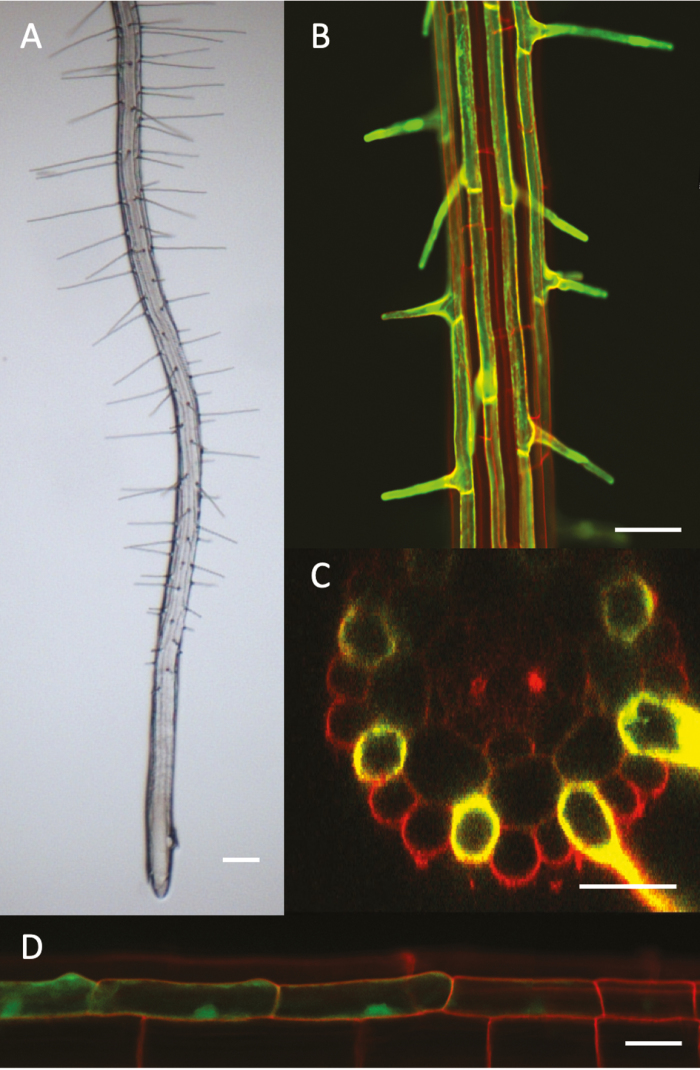
Arabidopsis root and root hairs. (A) Brightfield image of a root with root hairs. (B–D) Confocal images of a root hair-specific promoter::green fluorescent protein (GFP) line. (B) longitudinal section showing GFP in distinct root hair cell files; (C) cross-section showing root hair cell locations marked by GFP; (D) longitudinal section through an epidermal cell file showing GFP expression in two consecutive developmental stages: at the end of the determination phase (at the right) and in the root hair initiation phase (from the middle to the left). Scale bar: 200 μm (A), 50 μm (B), 20 μm (C, D).

**Fig. 2.**
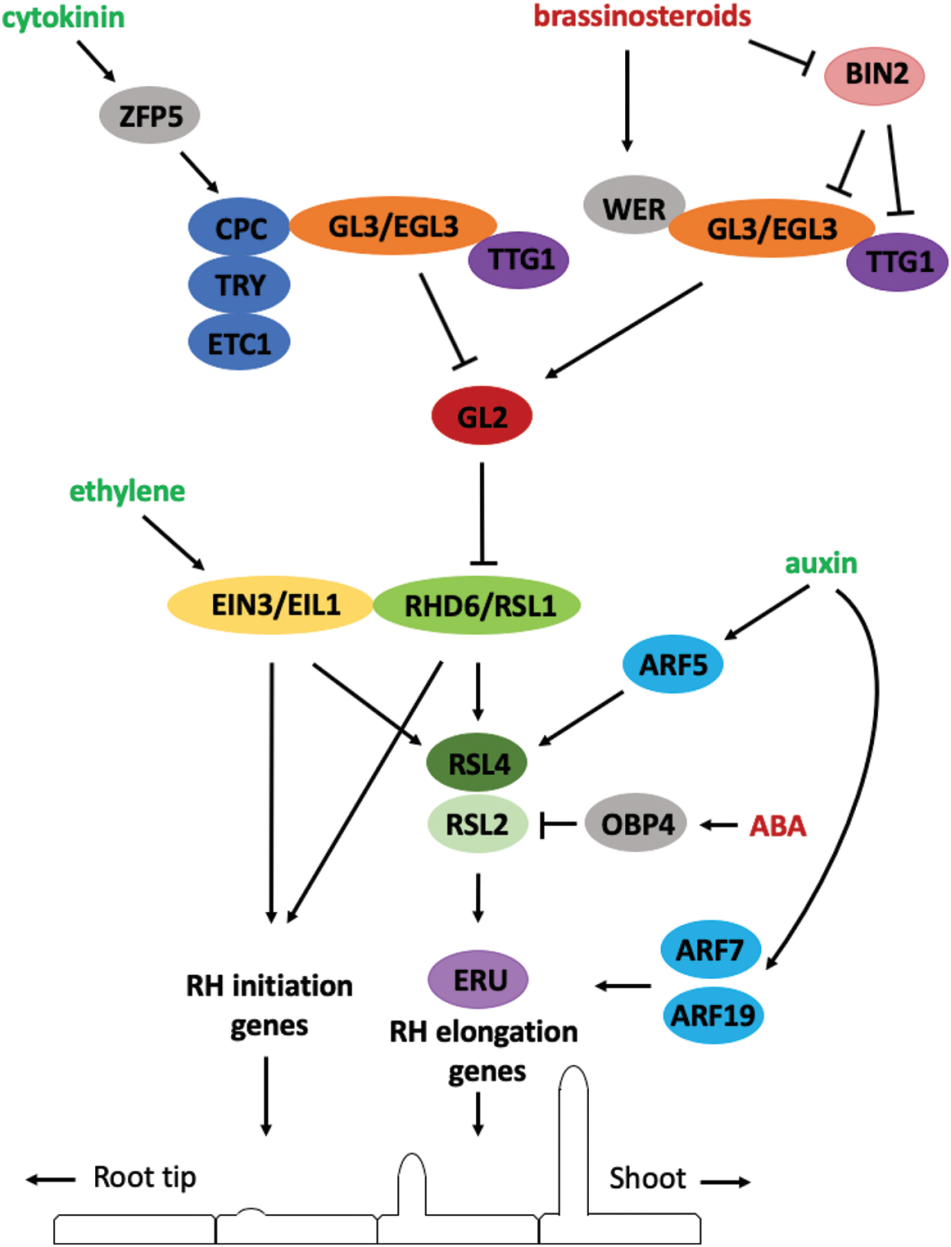
Model of the molecular and hormonal pathway for root hair development. Hormones stimulating root hair growth are shown in green, those repressing are shown in red. Root tip is at the left side in the schematic longitudinal section through a single epidermal cell layer with consecutive root hair developmental phases. ARF5/7/19, AUXIN RESPONSE FACTOR 5/7/19; BIN2, BRASSINOSTEROID-INSENSITIVE 2; CPC, CAPRICE; EGL3, ENHANCER OF GLABRA 3; EIN3, ETHYLENE INSENSITIVE 3; EIL1, ETHYLENE INSENSITIVE 3-LIKE 1; ETC1, ENHANCER OF TRY AND CPC1; GL2/3, GLABRA2/3; OBP4, OBF BINDING PROTEIN 4; RHD6, ROOT HAIR DEFECTIVE 6; RSL1–4, ROOT HAIR DEFECTIVE 6-LIKE 1–4; TRY, TRYPTICHON; TTG1, TRANSPARENT TESTA GLABRA 1; WER, WEREWOLF; ZFP5, ZINC FINGER PROTEIN 5.

Root hair development starts with the determination of whether an epidermal cell becomes a root hair (H; trichoblast) or non-root hair (N; atrichoblast) cell, giving rise to distinct hair and non-hair cell files in the Arabidopsis root (Fig. 1; Dolan *et al.*, 1994). Epidermal cell fate determination depends on a position-dependent signal originating from the underlying cortical cells. Signal perception in epidermal cells that overlie two cortical cells triggers a transcription factor cascade that ultimately inhibits the expression of GLABRA2 (GL2), a core non-root hair-determining transcription factor. As a result, ROOT HAIR DEFECTIVE 6 (RHD6) expression is initiated, leading to the initiation of a root hair bulge (Fig. 2). This bulge then begins tip growth before ultimately maturing. Root hair initiation and tip growth are each characterized by distinct yet interconnected molecular pathways (extensively reviewed in Balcerowicz *et al.*, 2015; Schoenaers *et al.*, 2017).

This genetic framework, which guides root hair development (Fig. 2), is heavily controlled by hormonal cues. In particular the influence of auxin and ethylene is well documented. These hormonal signals are key to the plant’s ability to dynamically regulate root hair form and function in response to the changing properties of the surrounding soil environment. In addition, crosstalk exists between several hormone classes (Wang and Irving, 2011; Zhang *et al.*, 2016; Liu *et al.*, 2017; Zhang *et al.*, 2018).

### Auxin

**Fig. 3.**
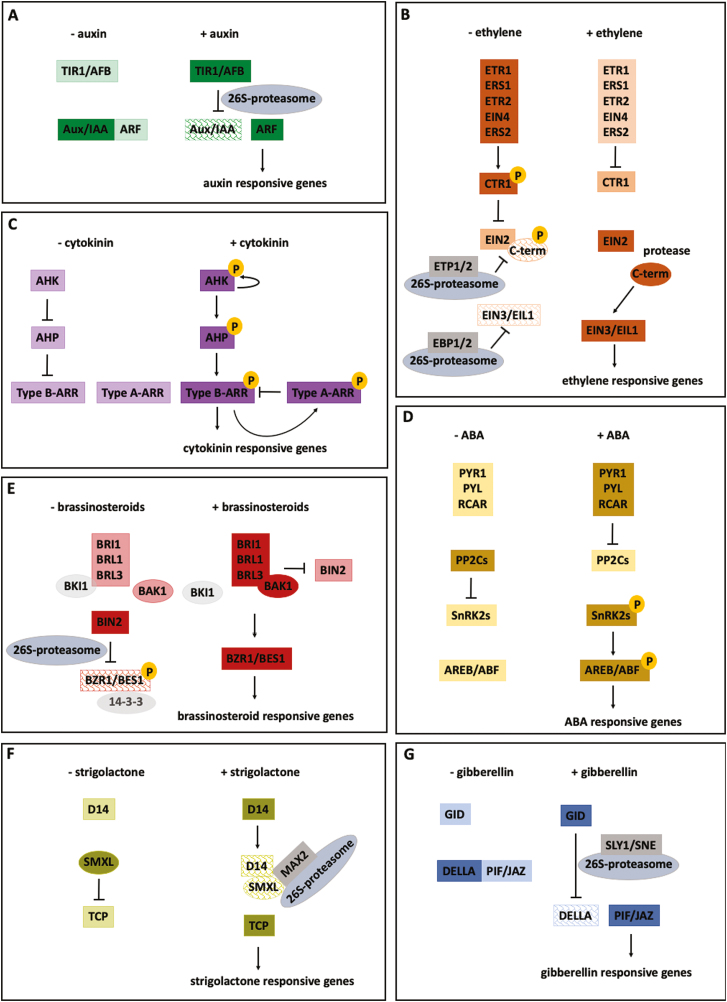
Models for hormone-responsive transcription. Simplified models describing how auxin (A), ethylene (B), cytokinin (C), abscisic acid (D), brassinosteroid (E), strigolactones (F), and gibberellin (G) regulate transcription of their responsive genes. Left: absence of hormone, and right: presence of hormone. Active proteins are represented by dark colour, inactive proteins by pale colour, and shingled colour indicates the proteins are targeted for ubiquitination and subsequent degradation by the 26S-proteasome.

Auxin is a well-characterized hormone that influences many plant developmental processes and acts as a positive regulator of root hair development (Paque and Weijers, 2016). Most auxin responses occur as a result of transcriptional and translational changes. This signalling pathway is known as the canonical pathway. To the contrary, other responses occur too fast to be dependent on nuclear transcription. These non-canonical pathways (Kubeš and Napier, 2019) will not be discussed in this review. The TRANSPORT INHIBITOR RESISTANT1/AUXIN SIGNALING F-BOX (TIR1/AFB) receptor complex that binds to auxin is the substrate-recognition subunit of the SUPPRESSOR OF KINETOCHORE PROTEIN 1 (SKP1)/CULLIN1/F-Box (SCF) E3 ubiquitin ligase complex. Auxin–TIR1 binding promotes the interaction between TIR1/AFB and AUXIN/INDOLE-3-ACETIC ACID (Aux/IAA) co-repressor proteins, which triggers the polyubiquitination and degradation of these Aux/IAAs. The latter causes dissociation of Aux/IAAs from auxin response factors (ARFs), allowing these transcription factors to regulate gene transcription (Fig. 3A; Teale *et al.*, 2006; Weijers and Wagner, 2016; Ma *et al.*, 2018).

Overexpression of ARF5, 7, and 8 enhances root hair growth (Mangano *et al.*, 2017), while root hair-specific overexpression of ARF1–4, 9–11, and 16 inhibits root hair growth, indicating that root hair development depends on the relative transcriptional activity of ARFs, which function as activators (aARFs) or repressors (rARFs) of gene transcription (Choi *et al.*, 2018). Several auxin-signalling mutants, including *aux1*, *axr1*, *axr2/iaa7*, *arf5*, *arf7/arf19*, *axr3/iaa17*, *slr/iaa14*, and *iaa28* have altered root hair development (Masucci and Schiefelbein, 1996; Rahman *et al.*, 2002; Okushima *et al.*, 2005; Li *et al.*, 2016), and supplementation or overproduction of auxin [e.g. gain-of-function mutant SHORT HYPOCOTYL 2 (SHY2/IAA3] leads to longer root hairs (Pitts *et al.*, 1998; Knox *et al.*, 2003). In addition, loss- and gain-of-function mutants of DIOXYGENASE FOR AUXIN OXIDATION 1 (DAO1), a protein that plays a major role in auxin oxidation and thus in regulating the cellular homeostasis of auxin, display altered root hair length (Porco *et al.*, 2016). Together, these findings clearly illustrate the pivotal role of auxin in controlling root hair morphogenesis.

Masucci and Schiefelbein (1996) have shown that auxin is capable of restoring root hair growth even in the hairless *rhd6* mutant, which lacks the core root hair-initiating basic helix–loop–helix (bHLH) transcription factor RHD6 (Fig. 2). According to Yi *et al.* (2010), this occurs through the induction of the bHLH transcription factor ROOT HAIR DEFECTIVE 6-LIKE4 (RSL4), which acts downstream of RHD6. As such, *RSL4* is considered to be the first auxin-responsive gene in the timeline of root hair morphogenesis. During root hair initiation, a 4 h-long *RSL4* expression pulse determines final root hair length. As shown in Fig. 2, auxin induces *RSL4* expression through a direct interaction of ARF5 with the *RSL4* promoter (Mangano *et al.*, 2017). Surprisingly, to date, none of the genes functioning upstream of *RSL4* has been shown to be auxin regulated, suggesting that auxin is not involved in early root hair development. Conversely, it has been shown that auxin is able to directly regulate the expression of genes downstream of *RSL4*. For instance, ARF7 and ARF19 directly interact with the promoter sequence of the *RSL4*-target gene *ERULUS*, a trichoblast-specific receptor-like kinase and core regulator of the root hair tip growth process (Schoenaers *et al.*, 2018). The relative importance of direct (through ARFs) and indirect (through *RSL4*) auxin-controlled gene expression remains to be investigated.

### Ethylene

The gaseous hormone ethylene is a positive regulator of root hair development, and frequently found to functionally interact with auxin signalling (Dubois *et al.*, 2018). A family of five receptors perceives ethylene, ETHYLENE RESPONSE 1 (ETR1), ETR2, ETHYLENE RESPONSE SENSOR 1 (ERS1), ERS2, and ETHYLENE INSENSITIVE 4 (EIN4), which are functionally redundant (Dong *et al.*, 2010; Ju and Chang, 2015). In the absence of ethylene, the ethylene receptors phosphorylate and activate the Ser/Thr kinase CONSTITUTIVE TRIPLE RESPONSE 1 (CTR1). Subsequent phosphorylation of ETHYLENE INSENSITIVE 2 (EIN2) by CTR1 leads to the activation of EIN2 TARGETTING PROTEIN 1 and 2 (ETP1/2), which mediates ubiquitination-dependent degradation of the C-terminal domain of EIN2. As C-terminal EIN2 is required for stabilization of ETHYLENE INSENSITIVE3 (EIN3) and EIN3-LIKE 1 (EIL1) transcription factors in the nucleus, EIN3 and EIL1 remain inactive and are targeted for proteasome-dependent degradation, which requires EIN3 BINDING F-BOX 1 and 2 (EBF1/2) when ethylene is absent (Binder *et al.*, 2007). The binding of ethylene inactivates the receptor signalling. As a consequence, EIN2 is not phosphorylated by CTR1, and a cytoplasmic C-end part is proteolytically cleaved off. The latter then moves to the nucleus, stabilizes EIN3 and EIL1, and facilitates expression of ethylene-responsive (ERF) genes (Fig. 3B; Ju and Chang, 2015; Moniuszko, 2015; Shakeel *et al.*, 2015).

Loss of ETR1 and EIN2 function (leading to ethylene insensitivity) or blocking of ethylene biosynthesis inhibits root hair growth (Masucci and Schiefelbein, 1996; Rahman *et al.*, 2002). Supplementation of the ethylene precursor aminocyclopropane carboxylic acid (ACC), on the other hand, stimulates root hair growth. Importantly, ACC supplementation also induces the formation of ectopic root hairs (Pitts *et al.*, 1998). Together, these data show that ethylene is a positive regulator of root hair development that, unlike auxin, also regulates the epidermal cell fate determination pathway.

Ethylene stimulates the expression of different key root hair genes such as *RHD6*, *RSL4*, and *RSL2*, which regulate the later stages of root hair initiation and tip growth (Masucci and Schiefelbein, 1994; Yi *et al.*, 2010, Zhang *et al.*, 2016). Feng *et al.* (2017) showed that EIN3 physically interacts with RHD6, and that both transcription factors coactivate the expression of *RSL4* in the presence of ethylene (Fig. 2). RHD6 forms a transcriptional complex with RSL1, another bHLH transcription factor of the RSL family that seems to be an upstream regulator of *RSL2* (Pires *et al.*, 2013). Together with the complex EIN3–EIL1, they are currently considered to be key regulators of root hair initiation and elongation (Feng *et al.*, 2017). Whether EIN3 also interacts with RSL1 and whether EIL1 acts similarly is currently unknown. Furthermore, ethylene signalling seems to go specifically through *RHD6* and *RSL1* because root hair initiation cannot be rescued by ethylene in the *rhd6 rsl1* double mutant. Transcriptional analysis revealed the genes that are regulated by EIN3–EIL1 and RHD6–RSL1, which potentially control root hair initiation and/or elongation (Feng *et al.*, 2017). Further research is needed to better understand ethylene-controlled root hair morphogenesis and where and how auxin and ethylene intersect in the root hair formation pathway.

### Cytokinin

Cytokinins control the cell cycle and several plant developmental processes (Werner and Schmülling, 2009; Bishopp *et al.*, 2011; Kushwah *et al.*, 2011; Muraro *et al.*, 2011; Su *et al.*, 2011).

There are few reports regarding the role of cytokinins in regulating root hair development. Recent findings have shed new light on the molecular basis of the cytokinin signalling pathway. Cytokinin binds to His kinase-like proteins such as ARABIDOPSIS HISTIDINE KINASE 2/3/4 (AHK2/AHK3/AHK4) triggering their autophosphorylation. Subsequent phosphate-group transfer towards an ARABIDOPSIS histidine phosphotransfer protein (AHP) leads to AHP nuclear translocation and the activation of type-B Arabidopsis response regulators (ARRs), which in turn induce cytokinin-mediated gene transcription. Finally, cytokinin signalling is subject to negative feedback regulation by expression of type-A ARRs, which are negative regulators of type-B ARRs (Fig. 3C; Hutchison and Kieber, 2002; Hwang *et al.*, 2012; Kieber and Schaller, 2014; Huang *et al.*, 2018*b*).

The role of cytokinin in root hair development remained unclear until the transcription factor ZINC FINGER PROTEIN5 (ZFP5) was discovered. Loss-of-function *zfp5* mutants exhibit a short root hair phenotype, caused by slower growth, which cannot be rescued by cytokinin addition. In wild-type plants, however, cytokinin supplementation induces an increase in final root hair length, while lowering endogenous cytokinin levels results in a short root hair phenotype. ZFP5, which is cytokinin-inducible, seems to lie at the base of cytokinin-regulated root hair morphogenesis and directly promotes the expression of *CPC*, a positive root hair regulator (Fig. 2; An *et al.*, 2012; Zhang *et al.*, 2016). At the moment, it needs to be investigated whether other genes in the root hair developmental pathway are (direct) targets of cytokinin signalling and whether cytokinin acts through ZFP5 only.

### Abscisic acid

Abscisic acid (ABA) controls seed dormancy, germination (Kermode, 2005), inhibition of root growth, and the response to abiotic stresses such as drought and salinity (Wang *et al.*, 2007; Luo *et al.*, 2014; Lombardo and Lamattina, 2018). ABA is perceived by cytosolic receptors, designated as PYRABACTIN RESISTANCE1 (PYR1), PYR1-LIKE (PYL), or REGULATORY COMPONENTS OF ABA RECEPTOR (RCAR). Under the absence or low levels of ABA, protein phosphatases of type 2C (PP2Cs) dephosphorylate SNF1-related protein kinases (SnRKs), thereby reducing SnRK2 kinase activity and inhibiting ABA-induced gene transcription. Under higher ABA concentrations, the RCAR receptors mediate the inactivation of the PP2Cs. As a consequence, SnRK2s activate several transcription factors from the ABA RESPONSIVE ELEMENT (ABRE) BINDING proteins (AREBs)/ABRE BINDING FACTORs (ABFs), initiating ABA-induced gene expression (Fig. 3D; Ma *et al.*, 2009; Fujii *et al.*, 2009; Melcher *et al.*, 2009; Li *et al.*, 2018).

Abscisic acid is a negative regulator of root hair growth since supplementation results in reduced root hair growth, a response that is absent in ABA-insensitive mutants. Recent work shows that ABA controls root hair growth by inhibiting the expression of the key root hair transcription factor RSL2 through the up-regulation of OBF BINDING PROTEIN4 (OBP4; Fig. 2). This transcription factor in turn binds to the *RSL2* promoter sequence and negatively regulates its transcription. In line with the latter, root hairs of *rsl2* were found to be irresponsive to ABA treatment (Rymen *et al.*, 2017).

### Brassinosteroids

Brassinosteroids (BR) are endogenous plant hormones that are involved in multiple developmental processes (Müssig *et al.*, 2003; Kuppusamy *et al.*, 2009; Clouse, 2011; Wei and Li, 2016). They are perceived by the receptor kinases BRASSINOSTEROID-INSENSITIVE 1 (BRI1) and its paralogs BRI1-LIKE 1 (BRL1) and BRI1-LIKE 3 (BRL3). BRI1 is expressed throughout the root, while BRL1 and BRL3 are mainly found in the stem cell niche (Planas-Riverola *et al.*, 2019). BRI1–BR association leads to BRI1 binding to BRI1-ASSOCIATED RECEPTOR KINASE (BAK1). This BRI1–BAKI heterodimer complex then initiates a phosphorylation relay cascade involving BRI1 KINASE INHIBITOR (BKI1) dissociation, which finally ends with dephosphorylation and activation of the transcription factors BRASSINAZOLE RESISTANT 1 (BZR1) and BRI1-EMS-SUPPRESSOR (BES1), allowing them to regulate BR-induced gene transcription. In the absence of BR, the kinase BRASSINOSTEROID-INSENSITIVE 2 (BIN2) phosphorylates BZR1/BES1, which inactivates them and promotes binding to 14-3-3 proteins. This interaction leads to their cytoplasmic retention and degradation (Fig. 3E; Zhu *et al.*, 2013; Nolan *et al.*, 2017; Yu *et al.*, 2018; Planas-Riverola *et al.*, 2019).

BRs seem essential for position-dependent epidermal cell fate determination in roots (Wei and Li, 2016). BR-deficient mutants display ectopic root hair formation, whereas BR-signal-enhanced mutants produce fewer root hairs. In line with this, treatment with brassinolide or bikinin produced fewer root hairs, while brassinazole, a BR-synthesis inhibitor, led to a change in epidermal cell fate determination resulting in the formation of more trichoblasts and thus more root hairs (Cheng *et al.*, 2014). Kuppusamy *et al.* (2009) found that BRs positively regulate the expression of *WER* and *GL2*, both negative regulators of early root hair development (Fig. 2). Root hair formation is determined by transcription factor complex formation and lateral communication between H- and N-cells (Bernhardt *et al.*, 2005; Savage *et al.*, 2008). Cheng *et al.* (2014) have demonstrated that BRs interfere with this process, leading to aberrant cell-specific GL2 and WER activity. At low BR concentrations, the formation and activity of the transcription factor complex (WER–GL3/EGL3–TTG1) is inhibited in both H- and N-cells since *WER* expression is lower and the kinase BIN2 phosphorylates EGL3 and TTG1, which reduces the functionality of the transcription factor complex. This suppresses *GL2* expression and thus results in formation of more root hairs as hair cell fate is promoted. At high BR concentrations, *WER* expression is increased in both N- and H-cells, the WER–GL3/EGL3–TTG1 transcription factor complex is fully functional (since BIN2 is not active), promoting *GL2* expression and determination of the non-hair cell fate in all epidermal cells (Cheng *et al.*, 2014; Wei and Li, 2016).

### Strigolactones

Strigolactones (SLs) were recently classified as a class of phytohormones (Brewer *et al.*, 2013). DWARF 14 (D14) receptor proteins perceive strigolactones, leading to a conformational change and subsequent signal transduction. Similar to other hormone-perception mechanisms, SL signal transduction depends on the initial ubiquitin-dependent degradation of SL-suppressor proteins (e.g. SUPPRESSOR OF MORE AXILLARY GROWTH 2 (MAX2)-LIKE family proteins; SMXLs). SMXL degradation allows downstream signalling through TCP-domain transcription factors (Fig. 3F; Machin *et al.*, 2020).

Kapulnik *et al.* (2011*a*,*b*) have described that supplementation with synthetic SL (GR24) enhances root hair growth. This positive effect of SLs depends, at least in part, on MAX2 F-box proteins that function in ubiquitin-dependent protein degradation. MAX2/3/4 loss-of-function plants display a short root hair phenotype. However, contrary to *max3* and *max4* mutants, the *max2* root hair length can only be restored at high GR24 concentrations. These data highlight an emerging role for SLs in controlling root hair morphogenesis.

### Gibberellin

Among other things, gibberellins (GA) control cell elongation, root apical meristem (RAM) growth, and the formation of lateral roots (Ogawa *et al.*, 2003; Tyler *et al.*, 2004; Davière and Achard, 2013, 2016; Hu *et al.*, 2018), yet little is currently known on GA involvement in root hair development. GA signal perception works through three orthologs of GIBBERELLIN INSENSITIVE DWARF 1 (GID1A–C), soluble nuclear receptors, which consist of a GA-binding pocket and a flexible N-terminal extension. GID1–GA binding leads to a GID1 conformational change that enables subsequent interaction with DELLA repressor proteins, leading to the formation of a GA–GID1–DELLA complex. Upon GA–GID1–DELLA complex formation, the DELLA proteins undergo small conformational changes that enhance recognition with the F-box proteins SLEEPY 1 (SLY1) and SNEEZY (SNE/SLY2). This promotes DELLA ubiquitination and subsequent destruction of DELLAs by the 26S proteasome. As a result, the transcription factors (PIFs, JAZs) that were inactivated by DELLAs become de-repressed and GA-induced signal transduction is initiated (Fig. 3G; Davière and Achard, 2013).

There are no reports on direct root hair growth responses towards GA in Arabidopsis. Nevertheless, Devlin and Brown (1969) have shown that GA has a positive effect on the elongation rate of *Agrostis alba* root hairs. A similar effect was seen in *Datura innoxia*, where exogenous GA resulted in more hairy roots and increased root hair elongation (Ohkawa *et al.*, 1989). How GA feeds into the genetic root hair growth framework needs further investigation.

## The complex crosstalk among phytohormones during root hair development

Hormonal crosstalk is instrumental in plant development. The root hair developmental pathway is fundamentally controlled by input from several hormonal classes. Hence, understanding how hormone signalling pathways are interconnected is a crucial step towards building an integrated view of root hair morphogenesis.

### Auxin and ethylene

A large effort has gone into understanding the heavily interconnected auxin and ethylene signalling pathways during root hair growth (Vanstraelen and Benková, 2012; Muday *et al.*, 2012; Robles *et al.*, 2013; Lee and Cho, 2013; Qin and Huang, 2018). A functional interaction is illustrated by the finding that auxin biosynthesis is up-regulated by ethylene in the root tip and during polar root hair initiation (Stepanova *et al.*, 2005, 2007; Růzicka *et al.*, 2007; Swarup *et al.*, 2007; Ikeda *et al.*, 2009). Vice versa, Strader *et al.* (2010) demonstrated that the *aux1* mutation suppresses the long hair phenotype of the *eto1* mutant, suggesting that ethylene needs auxin transport during the regulation of some phases of root hair development. In addition, the *rhd6* root hair-less phenotype can be rescued by both auxin and ACC (Masucci and Schiefelbein, 1994), showing that both hormones can influence root hair development downstream of *RHD6*-controlled gene transcription.

The importance of both auxin and ethylene signalling is further illustrated by the fact that root hairs are defective in the *aux1 ein2* double mutant (Rahman *et al.*, 2002) and that transcriptome analysis identified a large group of common root hair genes that are both up-regulated by auxin and ethylene supplementation (Bruex *et al.*, 2012). The nature of auxin–ethylene crosstalk is, however, complex. Hence, auxin and ethylene signalling are not per definition mutually interdependent. For instance, the phenotype of *aux1 etr1* double mutants can be suppressed by administration of IAA, but not by ACC addition (Masucci and Schiefelbein, 1996). To the contrary, *ein3 eil1 rhd6 rsl1* quadruple mutant roots remain hairless after auxin application, suggesting that the link between ethylene and auxin signalling is not that straightforward after all during root hair morphogenesis (Feng *et al.*, 2017). Nonetheless, auxin–ethylene crosstalk is embedded throughout a variety of plant developmental processes (Muday *et al.*, 2012). In general, it is now believed that functional auxin signalling is required for optimal ethylene responses. In line with the latter, the *aux1* and *tir1* auxin mutants show reduced sensitivity to ethylene treatment. Vice versa, it was found that the expression of auxin homeostasis and polar transport genes is increased as a result of increased ethylene levels (Zemlyanskaya *et al.*, 2018).

Mechanistically, the interconnection of auxin and ethylene signalling can be explained by the presence of an EIN3 binding region in the *AUXIN RESISTANT 1* (*AUX1*) promoter (Zažímalová *et al.*, 2007). Similarly, ACC transport is promoted by LYSINE HISTIDINE TRANSPORTER 1 (LHT1), which in turn is under the control of auxin (Lewis *et al*, 2013). Moreover, the transcription of 1-AMINOCYCLOPROPANE-1-CARBOXYLATE SYNTHASE (ACS) genes is up-regulated by auxin in an ARF-dependent manner (Stepanova *et al.*, 2007). Auxin and ethylene seem to impinge on the same core root hair pathway, through RSL4 and RHD6, respectively, suggesting that both hormonal signals need to be considered in order to understand root hair growth.

### Cytokinin, auxin and ethylene

A functional auxin–cytokinin interaction during plant development is emerging. Surprisingly, cytokinin and auxin were found to interact in a synergistic or antagonistic manner during plant development. For instance, both hormones were found to positively regulate root/stem growth and flowering (Srivastava, 2002; Moubayidin *et al.*, 2013; El-Showk *et al.*, 2013; Schaller *et al.*, 2015; Liu *et al.*, 2017). Contrastingly, auxin and cytokinin have an antagonistic role in lateral root formation. On one hand, type-B ARR1 stimulates the expression of *SHY2*, which encodes IAA3 (Tian and Reed, 1999) and negatively influences auxin signalling. This indirectly causes degradation of PIN1 and thereby suppresses auxin transport (Dello Ioio *et al.*, 2008; Marhavý *et al.*, 2014), which leads to an antagonistic relationship between auxin and cytokinin. On the other hand, auxin can inhibit SHY2/IAA3 through degradation via the SCF complex, resulting in PIN expression and subsequent auxin transport. In addition, Schaller *et al.* (2015) have shown that auxin directly activates the transcription of *ARR7* and *ARR15*, both type-A ARRs known to be negative regulators of the cytokinin response. During early lateral root development auxin promotes the cytokinin inhibitor AHP6, which represses cytokinin signalling by modulating PIN1 and, as such, auxin distribution (Moreira *et al.*, 2013).

Cytokinin, together with ethylene, stimulates the abscission of different plant organs. In fact, the stability of ACC synthase can be increased by cytokinin, resulting in higher ethylene production (Chae *et al.*, 2003; Hansen *et al.*, 2009). For root hair development, however, the effect of cytokinin might be independent of auxin and ethylene, especially since cytokinin can rescue root hair elongation when auxin (*axr1*) and ethylene signalling (*etr1-1*) are disturbed (Zhang *et al.*, 2016). Furthermore, auxin and ethylene can stimulate root hair elongation when cytokinin is defective in an overexpression line of CKX2, a cytokinin oxidase (Zhang *et al.*, 2016). It is therefore plausible that several common genes in the root hair-forming network can be targets for auxin, ethylene, and cytokinin. This idea is strengthened by the observation that the phenotype of the *rhd6* mutant can be rescued by exogenous auxin and ethylene, but also by cytokinin (Masucci and Schiefelbein, 1994; Zhang *et al.*, 2016). Candidates downstream of RHD6 are RSL1/2/4 (Fig. 2). In addition, auxin regulates cytokinin biosynthesis (Nordström *et al.*, 2004) as the auxin response factors ARF5 and ARF7 control cytokinin biosynthesis by up-regulating *CYTOKININ RESPONSE FACTOR* (*CRF*) genes and cytokinin biosynthetic enzymes Liu *et al.* (2017). Furthermore, it has been demonstrated that cytokinin can regulate ethylene biosynthesis (El-Showk *et al.*, 2013), showing a complex interplay of cytokinin with other hormones and as such influencing root hair development.

### Strigolactones, auxin, and ethylene

Strigolactones have emerged as major players in regulating plant growth and development (Brewer *et al.*, 2013). Several studies have shown that SLs functionally interact with auxin transport through effects on PIN and TIR1 protein abundance (Kapulnik *et al.*, 2011*a*,*b*; Ruyter-Spira *et al.*, 2011; Mayzlish-Gati *et al.*, 2012; Machin *et al.*, 2020). These changes in auxin transport lead to changes in auxin levels, which can impact on root hair development. However, how exactly SLs regulate PIN abundances remains unclear.

Treatment of mutants showed that SL signalling is not required for ethylene-responses in root hairs and that SL responses need ethylene synthesis and signalling. In line with this, SL treatment induced ACC synthase genes, encoding key rate-determining enzymes in ethylene synthesis (Yamagami *et al.*, 2003). Besides, SL signalling is not required for auxin to elicit its root hair growth stimulating effect, while auxin perception is needed to some extent for the SL effect on root hairs. In addition, auxin and SL have an additive effect on root hair development. Treatment of *aux1-7 ein2-1* mutants, which are impaired in sensitivity towards auxin and ethylene, showed reduced sensitivity to SLs. These observations, together with the finding that SLs can regulate PIN abundances in an unknown manner, strongly suggest that there is intimate crosstalk between SLs, ethylene, and auxin in the regulation of root hair elongation. Evidence indicates that SL affects root hair elongation via up-regulation of the ethylene pathway, which in turn acts on root hair development and interacts with the auxin pathway. The latter is supported by the fact that SLs affect PIN abundances, auxin transport capacities, and thus cellular auxin (Kapulnik *et al.*, 2011*a*,*b*; Ruyter-Spira *et al.*, 2011; Mayzlish-Gati *et al.*, 2012; Machin *et al.*, 2020).

These descriptions clearly show that hormonal regulation of root/root hair development cannot be described by a linear signalling cascade. Instead, the mode of action of different hormones is integrated in a complex, interconnected network of which the developmental output is the result of crosstalk between the latter and direct and indirect effects. It is crucial to emphasize that there are still many outstanding questions regarding hormone interactions during root hair development.

## Influence of environmental stressors on root hair development and their interaction with hormones

Rhizosphere composition dramatically impacts root hair development. Nutrients and abiotic stressors are unevenly distributed throughout the soil. Hence, roots encounter heterogeneous conditions along their growth axis, forcing them to dynamically regulate root system architecture and, for example, root hair morphogenesis (Morris *et al.*, 2017; Shahzad and Amtmann, 2017).

### Environmental stressors affecting root hair development

Phosphorus (P_i_) is a crucial soil-immobile macronutrient that is taken up and recycled by the plant root. It is compartmentalized specifically to the top-soil, as its main source is plant detritus. Plants have evolved specialized mechanisms for P_i_ sensing, uptake and transport. Mechanistically, P_i_ status is sensed by SPX-domain-containing proteins. They act as cellular receptors for inositol pyrophosphate molecules (PP-InsP), a proxy for cellular P-content, and regulate P_i_ homeostasis (Puga *et al.*, 2017). SPX proteins negatively regulate PHOSPHATE STARVATION RESPONSE (PHR) transcription factor and its homologs PHR1-LIKE 1 (PHLs) (Puga *et al.*, 2014; Qi *et al.*, 2017). The SPX–PHR1 complex dissociates in the absence of PP-InsPs, releasing PHR1 to interact with the promoters of P_i_ starvation induced genes (PSI) (Bustos *et al.*, 2010; Wild *et al.*, 2016). Intriguingly, Ried *et al.* (2019, Preprint) recently described that InsP_8_/SPX-induced PHR1 oligomerization is required for PHR-mediated transcriptional regulation during P_i_ homeostasis. Choi *et al.* (2017) showed that sustained exposure to P_i_-deficient conditions results in a decrease of cellular ATP levels. The latter triggers PP-InsP generation and degradation by VIH1/2, two bifunctional inositol pyrophosphate kinases/phosphatases (Zhu *et al.*, 2019), allowing the plant to maintain its P_i_/ATP homeostasis and regulate the P_i_ starvation response.

**Fig. 4.**
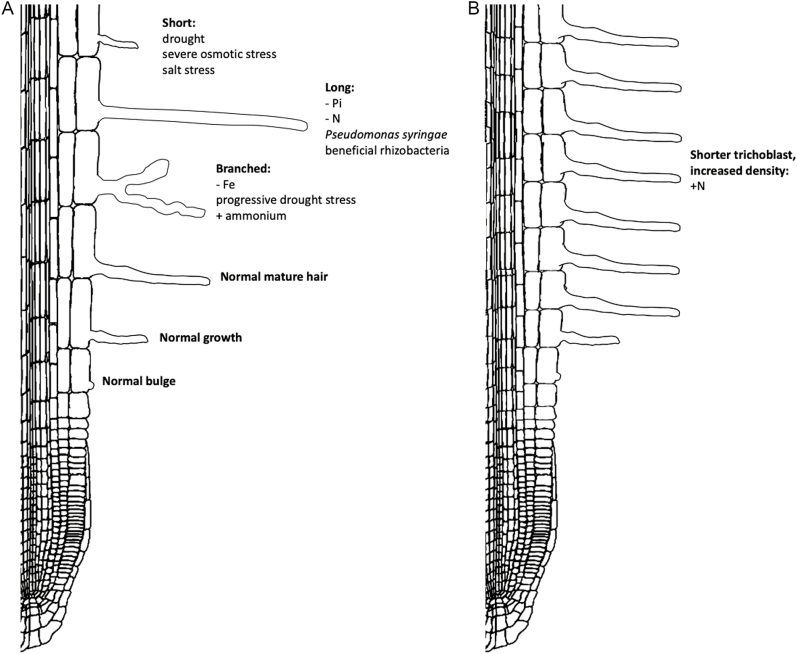
Effects of environmental conditions on Arabidopsis root hair development. Schematic representation of a longitundal section through an Arabidopsis root showing root hair development under normal conditions (A) and in altered root environments (B).

**Fig. 5.**
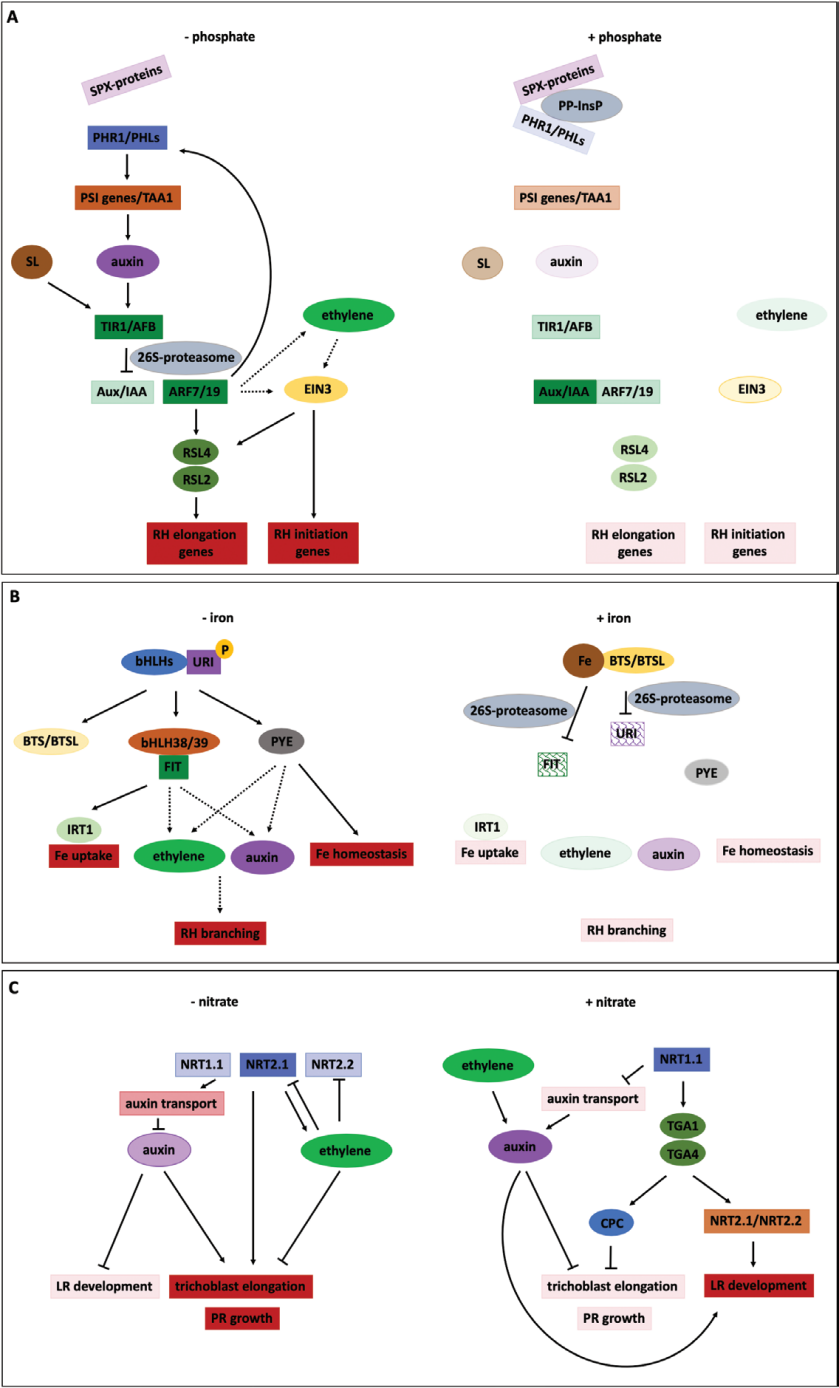
Models for signalling pathways downstream of phosphate, iron, and nitrate. Simplified models describing how phosphate (A), iron (B) and nitrate (C) deficiency or presence (left and right, respectively) regulate root hair development. Active proteins are represented in dark colour, inactive proteins are in pale colour, and shingled colour indicates the proteins are targeted for ubiquitination and subsequent degradation by the 26S-proteasome. For hormones, full colour and faded colour represent high and low concentrations. Dotted lines represent unknown relationships/interactions. ARF7/19, AUXIN RESPONSE FACTOR 7/19; Aux/IAA, AUXIN/INDOLE-3-ACETIC ACID; bHLHs, basic helix–loop–helix proteins; BTS, BRUTUS; BTSL, BRUTUS-LIKE; CPC, CAPRICE; EIN3, ETHYLENE INSENSITIVE 3; FIT, FER-LIKE IRON DEFICIENCY-INDUCED TRANSCRIPTION FACTOR; IRT1, IRON-REGULATED TRANSPORTER 1; LR, lateral root; NRT1.1/NRT2.1/NRT2.2, NITRATE TRANSPORTER 1.1/2.1/2.2; PHL, PHOSPHATE STARVATION RESPONSE 1-LIKE; PHR1, PHOSPHATE STARVATION RESPONSE 1; PR, primary root; PSI, phosphate starvation induced; PYE, POPEYE; RH, root hair; RSL2/4, ROOT HAIR DEFECTIVE 6-LIKE 2/4; SL, strigolactone; SPX-proteins, SPX-domain containing proteins; TAA1, TRYPTOPHAN AMINOTRANSFERASE OF ARABIDOPSIS 1; TGA1/4, TGACG SEQUENCE-SPECIFIC BINDING PROTEIN 1/4; TIR1/AFB, TRANSPORT INHIBITOR RESISTANT1/AUXIN SIGNALING F-BOX; URI, UPSTREAM REGULATOR OF IRT 1.

In low phosphate conditions the root is shorter, more lateral roots are formed, and root hairs grow much longer and denser (Fig. 4), facilitating top-soil foraging and increased nutrient uptake (Ma *et al.*, 2001; Koevoets *et al.*, 2016; Zhang *et al.*, 2018). These adaptations occur through local up-regulation of auxin biosynthesis (Bhosale *et al.*, 2018) and ARF7/19-induced *PHR1* expression, which promotes root hair growth (Huang *et al.*, 2018*a*). Recently Bhosale *et al.* (2018) found that P_i_ deficiency is first sensed at the root tip, causing up-regulation of TRYPTOPHAN AMINOTRANSFERASE OF ARABIDOPSIS 1 (TAA1), a crucial factor in the auxin biosynthetic pathway. AUX1-mediated auxin influx in the lateral root cap and adjacent epidermal cells is then required for increased auxin transport towards the epidermal root hair zone. Subsequently, an auxin-induced genetic cascade is initiated, which involves ARF19, RSL4, and RSL2, enhancing root hair elongation (Fig. 5A). Conversely, high phosphate conditions are able to strongly repress RSL4 expression and root hair growth. Mangano *et al.* (2018) revealed that when root hairs encounter two conflicting growth signals, i.e. high phosphate levels (inhibits root hair growth) and high auxin levels (enhances root hair growth), RSL2 plays an important role in restoring reactive oxygen species homeostasis and concomitantly in root hair growth recovery. The importance of RSL2 rather than RSL4 in the integration of conflicting growth signals is further illustrated by the finding that the *rsl4* mutant displays auxin-enhanced root hair length in either low or high P_i_ conditions, whereas in the presence of high phosphate, auxin supplementation of *rsl2* roots has no phenotypic effect.

According to Song *et al.* (2016), P_i_ starvation indirectly leads to ectopic root hair formation by elevation of EIN3 protein levels, in turn up-regulating root hair initiation and elongation genes (Fig. 5A). This was found in a dominant negative mutation of an ethylene receptor (ERS1). It is currently unknown whether the EIN3 level increases due to an increase in ethylene synthesis or ethylene-dependent auxin signalling. Besides auxin, also SLs play a role in P_i_-regulated root hair growth. Hence, SL concentrations increase under low P_i_ and nitrogen levels (Pérez-Torres *et al.*, 2008). In the presence of MAX2, the transcription of the auxin receptor TIR1 is stimulated. This promotes auxin signalling and results in an increase in root hair density and induction of PSI genes (Ma *et al.*, 2001; Chiou and Lin, 2011). Mayzlish-Gati *et al.* (2012) have shown that the supply of GR24 restores this response in SL-synthesis mutants, pointing to the importance of SL (Fig. 5A).

Iron (Fe) is a crucial micronutrient for plants as it is a key cofactor for various enzymatic reactions. However, Fe solubility is low in aerobic and neutral pH environments, leading to limited bioavailability and consequent Fe deficiency (Ivanov *et al.*, 2012). Arabidopsis roots alter Fe solubility by acidifying the rhizosphere through the action of the H^+^-ATPase AHA2. Iron is then reduced from ferric (Fe^3+^) to ferrous (Fe^2+^) iron by FERRIC REDUCTASE-OXIDASE 2 (FRO2), and the bivalent ion is then imported into root cells by IRON-REGULATED TRANSPORTER 1 (IRT1). In Arabidopsis, UPSTREAM REGULATOR OF IRT1 (URI) becomes phosphorylated under Fe deficiency and forms heterodimers with specific bHLH transcription factors to induce expression of many Fe-deficiency-induced genes, including BRUTUS (BTS), BRUTUS-LIKE 1 (BTSL1), POPEYE (PYE), and other class bHLHs. The latter induce the expression of the transcription factor FER-LIKE IRON DEFICIENCY-INDUCED TRANSCRIPTION FACTOR (FIT), which forms heterodimers with bHLH38/39/100/101 (Wang *et al.*, 2013) and regulates various genes involved in Fe uptake, such as IRT1 and FRO2 (Lan *et al.*, 2012; Kobayashi and Nishizawa, 2015). In parallel, PYE regulates the expression of genes that are involved in Fe homeostasis (Kim *et al.*, 2019). Under low Fe, BTS/BTSLs are inactive, but when Fe availability increases or is restored, Fe binding to the proteins activates their ubiquitin ligase activity (Kobayashi and Nishizawa, 2015) and they interact with the different transcription factor complexes, leading to their degradation (Kim *et al.*, 2019) (Fig. 5B).

Under low Fe conditions, rather than forming ectopic root hairs, plant roots increase their absorptive surface by inducing root hair branching (Müller and Schmidt, 2004; Lan *et al.*, 2012). Several lines of evidence suggest that both auxin and ethylene are involved in this response. The auxin-insensitive mutant *axr2-1* (Wilson *et al.*, 1990), mutated in *IAA7* (Nagpal *et al.*, 2000), also displays branched root hairs, as under Fe-deficient conditions. Transcriptome analysis showed that Fe deficiency causes differential expression of several genes involved in ethylene biosynthesis and auxin/ethylene signalling (Thimm *et al.*, 2001). It is plausible that these ethylene- and auxin-related genes are targets of the FIT and PYE transcription factors. The latter would explain the altered root hair morphology (due to changes in auxin responsiveness) and (low) percentage of ectopic root hair formation (by ethylene) (Fig. 5B). Importantly, Lan *et al.* (2011) reported that post-transcriptional and post-translational processes are also involved in the low Fe response.

Nitrogen is a major limiting mineral nutrient that is available in the soil as ammonium (NH_4_^+^) and nitrate (NO_3_^−^) (Forde, 2002). Besides being an essential nutrient, nitrate also functions as a signalling molecule regulating many different aspects of development, including root development (Walch-Liu *et al.*, 2006). This mobile nutrient is distributed by vertical water flow, known as leaching, and accumulates in deeper soils (Jobbágy and Jackson, 2001). Expectedly, low nitrate induces primary root growth yet inhibits lateral root development, enabling root systems to reach deeper soil layers (Bouguyon *et al.*, 2015; Araya *et al.*, 2016). In contrast, high nitrate conditions lead to slower primary root growth and an increase in root branching, which aids in the foraging for nutrients in that particular nitrate-rich soil patch or layer. When external nitrate concentrations are even higher, lateral root development is arrested in the early stages after emergence. Because of its high concentration in the soil, nitrate accumulates in the shoot and limits basipetal auxin fluxes, which results in lower root auxin and negative regulation of lateral root development (Walch-Liu *et al.*, 2006). Root hairs seem to play an important role in nitrate uptake, as root hair defective mutants accumulate significantly less nitrate than wild-types, while mutants with ectopic root hairs show an increase in nitrate content. Furthermore, root hairs seem important in detecting or sensing changes in nitrogen (Shin *et al.*, 2005). In addition, nitrate stimulates an increase in root hair density (Vatter *et al.*, 2015) by decreasing trichoblast cell lengths and not by inducing ectopic root hair formation, nor by stimulating root hair elongation (Canales *et al.*, 2017).

Two families of transporters participate in the uptake of nitrate in the root, NITRATE TRANSPORTER 1 and 2 (NRT1/2), where NRT1.1 and NRT2.1 are thought to be the most important (Okamoto *et al.*, 2003). To be in the active form as a nitrate transporter, NRT2.1 has to interact with NAR2.1 (also called NRT3.1) and form an NRT2.1–NAR2.1 hetero-oligomer (Yong *et al.*, 2010), whose activity is under post-translational control (Jacquot *et al.*, 2019, Preprint). Currently, NRT1.1 is the only known transporter for nitrate in Arabidopsis (Ho *et al.*, 2009; Giehl and von Wirén, 2015) and functions both as a transporter and as a receptor, called a transceptor. In line with the latter, NRT1-1 loss-of-function mutants failed to show an increase in nitrate-induced root hair density (Vatter *et al.*, 2015). Downstream of NRT1.1, two transcription factors, TGACG SEQUENCE-SPECIFIC BINDING PROTEIN 1 and 4 (TGA1/TGA4), play a redundant role in increasing the expression of *CPC*, which seems to be required for the reduction of trichoblast cell length and the subsequent increase in root hair density (Canales *et al.*, 2017). In addition, both transcription factors regulate the expression of NRT2.1 and NRT2.2, which are required for the induction of lateral root development but not for the reduction of cell elongation in the primary root (and resulting increase in root hair density). Furthermore, the nitrate transport function of NRT1.1 is needed for the increase in expression of the auxin receptor AFB3 in pericycle cells and its downstream target, NAC4 (Álvarez *et al.*, 2014; Vidal *et al.*, 2014). The involvement of auxin is further evidenced by the fact that NRT1.1 can facilitate the uptake of both nitrate and auxin. Consequently, in low nitrate conditions, auxin is taken up, while in higher nitrate conditions, nitrate outcompetes auxin leading to decreased auxin uptake through NRT1.1 and, consequently, auxin accumulation. This increased auxin level then affects lateral root development (Krouk *et al.*, 2010). Tian *et al.* (2009) have described a rapid rise in ethylene production upon exposure to high nitrate levels. It is known that increased ethylene concentrations lead to reduced root epidermal cell elongation and thus an increase in root hair density (Swarup *et al.*, 2007; Markakis *et al.*, 2013). The observation that both ethylene and auxin impact epidermal cell length could also explain the involvement of NRT1.1 in the shorter trichoblast cell length in high nitrate conditions (Fig. 5C).

In nitrate-deficient conditions, simultaneous *NRT1.1*, *NRT2.1*, and *NRT2.2* expression is required for maintaining root growth (Ye *et al.*, 2019). Zheng *et al.* (2013) have demonstrated that *NTR2.1* is strongly up-regulated in low nitrate conditions in an attempt to enhance nitrate uptake. In parallel, ethylene biosynthesis is up-regulated and dependent on NRT2.1. Surprisingly, ethylene reduced the expression of *NRT1.1* and *NRT2.1* and concomitantly the uptake of nitrate, which acts as a negative feedback loop (Fig. 5C; Tian *et al.*, 2009). No data on root hair responses are available.

Furthermore, Arabidopsis possesses six AMMONIUM TRANSPORTERS (AMT1.1–5 and AMT2; Yuan *et al.*, 2007). The expression of *AMT1.1* and *AMT2* is up-regulated in N-deprived roots (Sohlenkamp *et al.*, 2002; Lanquar *et al.*, 2009), and root hair elongation is increased in this condition (Bloch *et al.*, 2011). In contrast, ammonium was found to stimulate root hair branching, yet inhibit their elongation (Yang *et al.*, 2011).

During drought, plants are confronted with osmotic stress, causing cell dehydration and membrane/macromolecule damage (Lisar *et al.*, 2012). Primary root growth, lateral root formation, and root hair development are reduced upon osmotic stress, a process that is heavily controlled by ABA in a complex interacting network with cytokinin and other phytohormones (De Smet *et al.*, 2003; Nakashima *et al.*, 2014; Singh and Laxmi, 2015; Rowe *et al.*, 2016; Tiwari *et al.*, 2017). ABA stimulates the expression of the gene for ABSCISIC ACID INSENSITIVE4 (ABI4) transcription factor, among many other genes, which in turn inhibits PIN1 expression, leading to reduced polar auxin transport (Shkolnik-Inbar and Bar-Zvi, 2010) and thus lower auxin levels in the root. Besides having a direct inhibitory effect on root hair development, ABA could thus also act indirectly through changing the levels of other hormones. Under progressive drought stress, root hairs seemed to branch more often, which was also mediated by ABA and auxin, probably in a similar (concentration-dependent) manner (Bobrownyzky, 2016).

Increased salinity inhibits plant growth by creating osmotic stress and inhibiting photosynthesis in the shoot (Koevoets *et al.*, 2016). In addition, primary and lateral root growth and root hair development are suppressed during salt stress (Wang *et al.*, 2008; Mabrouk and Heikal, 2008). Salt exposure causes down-regulation of PIN genes and stabilization of AUXIN RESISTANT3 (AXR3)/IAA17, reducing polar auxin transport and inhibiting auxin signalling, respectively. The resulting reduction in auxin levels and signalling then negatively affects root hair development (Liu *et al.*, 2015).

Biotic factors, such as fungi, bacteria, insects, and pathogens can influence plant development. The main actors in the defence against such biotic factors are salicylic acid (SA), which offers biotroph resistance, and jasmonic acid (JA), which mediates necrotroph resistance (Takatsuji and Jiang, 2014). From the six major hormones discussed in this review, only ethylene and cytokinin positively interact with SA and JA and provoke the synthesis of pathogenesis-related proteins (Yang *et al.*, 2015). Pathogenic strains of *Pseudomonas syringae* inhibit primary root elongation and stimulate lateral root and root hair formation/elongation, which requires functional ethylene signalling and does not seem to be associated with changed auxin levels (Pecenková *et al.*, 2017). The same effect is noticeable in environments containing beneficial rhizobacteria (Pecenková *et al.*, 2017) and mycorrhizal, saprotrophic, and pathogenic fungi. In their presence, a peculiar clumped root phenotype is observed, marked by a short primary root and more or longer lateral roots (Casarrubia *et al.*, 2016). Unfortunately, the effect on root hair development has not been reported.

### Environmental stressors with no reported effect on root hair development

Temperature changes greatly impact on root functioning. For instance, seedling growth at 26–29 °C stimulates primary root elongation (Hanzawa *et al.*, 2013; Wang *et al.*, 2016; Martins *et al.*, 2017; Feraru *et al.*, 2019). Although both auxin and BR were reported to be involved in this response, the underlying hormonal mechanism is currently under debate. Yet, it was recently shown that enhanced primary root growth at higher temperatures coincided with increased auxin signalling. The latter is linked to the inhibition of PIN-LIKES6 (PILS6) proteins that limit nuclear auxin by sequestering auxin to the ER (Feraru *et al.*, 2019). The effect of high temperature exposure on root hair development has not been studied/reported to date.

Shibasaki *et al.* (2009) have shown that acute cold stress (4 °C) leads to inhibition of root elongation due to blocked basipetal auxin transport. The latter was caused by reduced intracellular trafficking of PIN2 and PIN3. After low temperature acclimation, the elongation rate recovers. It is plausible to think that cold-affected auxin concentrations regulate root hair development, but the exact effect of low temperatures on root hair development is still largely unexplored.

## Conclusion and future remarks

A large effort has gone into understanding the regulatory mechanisms controlling root hair development, with the aim of harnessing this knowledge to improve root functioning in future environmental conditions (e.g. nutrient depletion, water deprivation). The combination of state-of-the-art tools to study the molecular-genetic background of plant growth has led to a drastically improved understanding of how different plant hormones impinge on the root hair growth pathway.

In this review we have highlighted the general root hair developmental scheme and how hormones control this. Combined, it is clear that highly complex hormonal crosstalk and sensing of rhizosphere conditions lie at the base of root hair morphogenesis. At this stage, constructing an integrated view of the root hair signalling cascade, including hormonal and environmental control, is too optimistic. Yet, with the scientific community increasingly embracing the complexity and interconnectivity of the involved pathways, and harnessing the power of -omic datasets and hormone marker and sensor lines, we are closing in on deciphering the role of novel genes in the different phases of root hair development. Integration of all these data allows us to link the environmental changes to changes in hormone levels and finally to their effect on the (sub)cellular processes that actually guide root hair growth.
